# Long Noncoding RNA LEMD1-AS1 Increases LEMD1 Expression and Activates PI3K-AKT Pathway to Promote Metastasis in Oral Squamous Cell Carcinoma

**DOI:** 10.1155/2022/3543948

**Published:** 2022-08-09

**Authors:** Zaiye Li, Jie Wang, Jianjun Wu, Ning Li, Canhua Jiang

**Affiliations:** ^1^Department of Oral and Maxillofacial Surgery, Center of Stomatology, Xiangya Hospital, Central South University, Changsha, China; ^2^Research Center of Oral and Maxillofacial Tumor, Xiangya Hospital, Central South University, Changsha, China; ^3^Institute of Oral Cancer and Precancerous Lesions, Central South University, Changsha, China; ^4^State Key Laboratory of Oral Diseases, National Clinical Research Center for Oral Diseases, Chinese Academy of Medical Sciences Research Unit of Oral Carcinogenesis and Management, West China Hospital of Stomatology, Sichuan University, Chengdu, Sichuan 610041, China; ^5^Department of Immunology, Xiangya School of Medicine, Central South University, Changsha, China

## Abstract

**Background:**

The survival rate of oral squamous cell carcinoma (OSCC) is only 50% due to a high incidence of metastasis. Long noncoding RNAs (lncRNAs) play a crucial role in OSCC genesis and progression, although their potential role in the metastasis of OSCC remains unclear.

**Methods:**

The transcriptome of 5 metastatic and 5 nonmetastatic OSCC samples were assessed by RNA sequencing. The biological functions and regulatory mechanisms of LEMD1-AS1 in OSCC were explored by in vitro and in vivo assays.

**Results:**

We identified 487 differentially expressed mRNAs (DEmRNAs) and 1507 differentially expressed lncRNAs (DElncRNAs) in OSCC with cervical lymph node (LN) metastasis relative to the nonmetastatic samples. In addition, both LEMD1-AS1 and its cognate LEMD1 were up-regulated in metastatic OSCC compared to nonmetastatic OSCC. Gain-of-function, loss-of-function, and rescue experiments indicated that LEMD1-AS1 upregulated LEMD1 to increase OSCC migration and invasion in vitro and in vivo. Mechanistically, LEMD1-AS1 stabilized LEMD1 and increased its mRNA and protein levels, and consequently activated the PI3K-AKT signaling pathway to facilitate OSCC metastasis.

**Conclusions:**

We established the lncRNA-mRNA landscape of metastatic OSCC, which indicated that LEMD1-AS1 enhanced OSCC metastasis by stabilizing its antisense transcript LEMD1. Thus, LEMD1-AS1 is a potential biomarker for predicting metastasis, as well as a therapeutic target of OSCC.

## 1. Background

Oral squamous cell carcinoma (OSCC) is one of the most commonly diagnosed malignancies worldwide [1–3] and is characterized by a high incidence of local invasion and cervical lymph node (LN) metastasis. Despite recent advances in surgery, chemoradiotherapy, and other targeted therapies, the overall survival of OSCC patients is still only 50% due to the high metastasis rates [4, 5]. Therefore, it is essential to identify the underlying mechanisms of OSCC metastasis in order to develop novel effective therapies.

Long noncoding RNAs (lncRNAs) are noncoding transcripts more than 200 nucleotides in length [6, 7] and are classified into the antisense, intronic, bidirectional, intergenic, and overlapping types. lncRNAs regulate gene expression via chromatin modification, miRNA quenching, direct modulation of mRNA stability, transcription, and translation, as well as protein stability control [8, 9], and are involved in tumor initiation and progression. The antisense lncRNAs account for approximately 50–70% of all lncRNAs and can exert their function through *cis*- or *trans*-mechanisms [10]. The *cis*-acting antisense lncRNAs bind to genes in their vicinity, while the *trans*-lncRNAs modulate more distant genes on the same or even on different chromosomes. Furthermore, *cis*-antisense lncRNAs modulate gene expression at the pretranscriptional, transcriptional, and posttranscriptional levels through DNA–lncRNA, lncRNA–RNA, or protein–lncRNA interactions. lncRNA-RNA interactions in particular are common during cancer initiation and progression and involve hybridization of the sense and antisense sequences into RNA duplexes that regulate the posttranscriptional outcome. Zhao et al. reported that MACC1-AS1 promoted gastric cancer cell metabolic plasticity by stabilizing MACC1 mRNA [11]. In addition, lncRNA PXN-AS1-L acts as an oncogene in non-small-cell lung cancer (NSCLC) by increasing PXN expression [12]. Yuan et al. found that MUC5B-AS1 promoted lung adenocarcinoma metastasis by forming RNA-RNA duplex with MUC5B. However, little is known regarding the function of antisense lncRNAs in metastatic OSCC.

To this end, we performed next-generation sequencing analysis of human OSCC tissues in order to map the differentially expressed RNAs and establish a lncRNA-mRNA interaction network. Accordingly, we identified lncRNA LEMD1-AS1 and its target gene LEMD1, which acts as an oncogene in multiple cancers, and analyzed their biological role in OSCC via functional assays. Our findings provide new insights into OSCC metastasis and identify a novel biomarker for prognostic prediction and targeted therapy.

## 2. Results

### 2.1. Overview of RNA Sequencing Data

**Figure 1:**
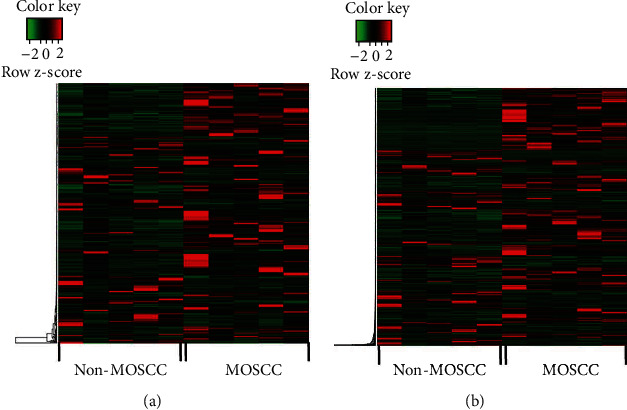
Heatmap of differentially expressed RNAs between metastatic OSCC and nonmetastatic OSCC. (a) mRNAs. (b) lncRNAs.

All raw data had been uploaded in GEO database (GSE145272, https://www.ncbi.nlm.nih.gov/geo/query/acc.cgi?acc=GSE145272). A total of 487 differentially expressed mRNAs (DEmRNAs) (319 upregulated and 168 downregulated) and 1507 differentially expressed lncRNAs (DElncRNAs) (971 upregulated and 536 downregulated) were identified in RNA-seq data using |log2 fold change (FC)| > 1.0 and *P* value < 0.05 as the thresholds (Figure 1). The functional enrichment analysis (Additional Figure 1A) showed that 487 DEmRNAs were enriched in 520 biological process (BP), 13 cellular component (CC), and 21 molecular function (MF) terms, including cell-cell adhesion, receptor complex, channel complex, channel activity, and receptor activity. KEGG pathway analysis (Additional Figure 1B) indicated enrichment of 243 pathways, including the cAMP signaling pathway, calcium signal pathway, cytokine-cytokine receptor interaction, and cell adhesion molecules (CAMs).

### 2.2. DElncRNA-DEmRNA Interaction Network

Potential interactions between the DElncRNAs and DEmRNAs were predicted using the LncTar software and correlated via R software. The interacting pairs were screened using cor ≠ 0 and *P* value < 0.05 as the thresholds. As shown in Additional Figure 2, 132 *cis*-regulation pairs and 165994 *trans*-regulation pairs were identified.

### 2.3. Validation of Dysregulated RNAs

**Figure 2:**
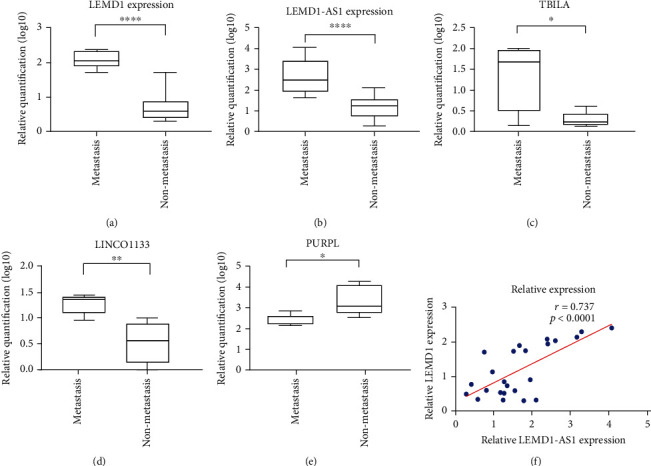
Validation of DERNA expression level in 10 OSCC samples included in next generation sequencing. (a) LEMD1. (b) LEMD1-AS1. (c) TBILA. (d) LINC01133. (e) PURPL. (f) Correlation between LEMD1-AS1 and LEMD1 mRNA expression in 24 OSCC tissues. ^∗^*P* < 0.05;  ^∗∗^*P* < 0.01;  ^∗∗∗^*P* < 0.001; *P* < 0.0001.

To validate the RNA-seq data, we randomly selected five DERNAs (LEMD1-AS1, LEMD1, TBILA, LINC01133, and PURPL) from the top 50 DERNAs identified in 10 samples by qPCR. LEMD1-AS1, LEMD1, TBILA, and LINC01133 were significantly upregulated in metastatic versus nonmetastatic OSCC while PURPL was downregulated in the former (Figure 2).

### 2.4. LEMD1-AS1 and LEMD1 Are Overexpressed in Human OSCC Tissues and Cell Lines

Since both LEMD1-AS1 and LEMD1 were upregulated in the metastatic OSCC samples relative to the nonmetastatic samples, we hypothesized that LEMD1-AS1 promotes OSCC metastasis by upregulating its predicted target LEMD1. To confirm our hypothesis, we detected the expression levels of both in additional OSCC samples by qRT-PCR. Consistent with the bioinformatics results, the metastatic tumors expressed higher levels of both LEMD1-AS1 and LEMD1, which showed a significant positive correlation (Figure 2(f)). In addition, the metastatic OSCC cell lines UM1, OSC19, and CAL27 showed significantly higher LEMD1-AS1 levels compared to the nonmetastatic UM2 and OSC3 cells (Additional Figure 3). Taken together, the LEMD1-AS1 and LEMD1 interaction is prometastatic in OSCC.

### 2.5. LEMD1-AS1 Promoted Migration of OSCC Cells via LEMD1

**Figure 3:**
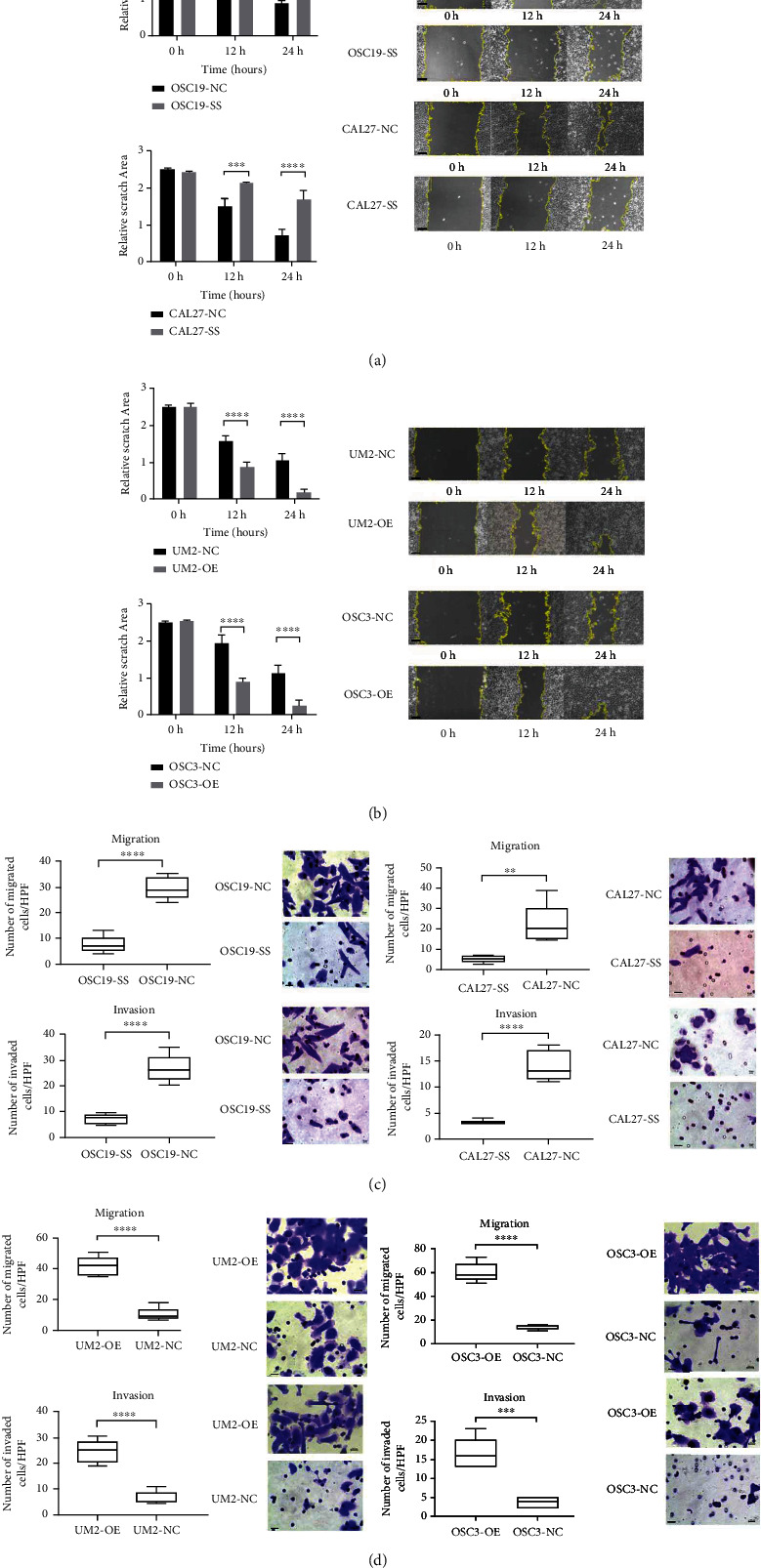
Wound healing assay showed that LEMD1-AS1 enhanced OSCC cells migration. The scope adumbrated by yellow line indicated the empty area calculated by ImageJ software. Data exhibited the mean of five biological replicates ± SE. (a) Downregulating LEMD1-AS1 expression significantly inhibited cell migration and invasion in OSC19 and CAL27 cells. (b) Upregulating LEMD1-AS1 expression highly promoted migration and invasion of UM2 and OSC3. (c) Transwell assay showed that up-regulating LEMD1-AS1 stimulated migration and invasion ability, while silencing LEMD1-AS1 repressed. The effect of LEMD1-AS1 in OSC19 and CAL27(C), UM2 and OSC3 (d). SS: LEMD1-AS1 Smart Silencer; NC: normal control. OE: LEMD1-AS1-overexpressing. Scale bar in wound healing assay = 100 *μ*m. Scale bar in transwell assay = 20 *μ*m.

**Figure 4:**
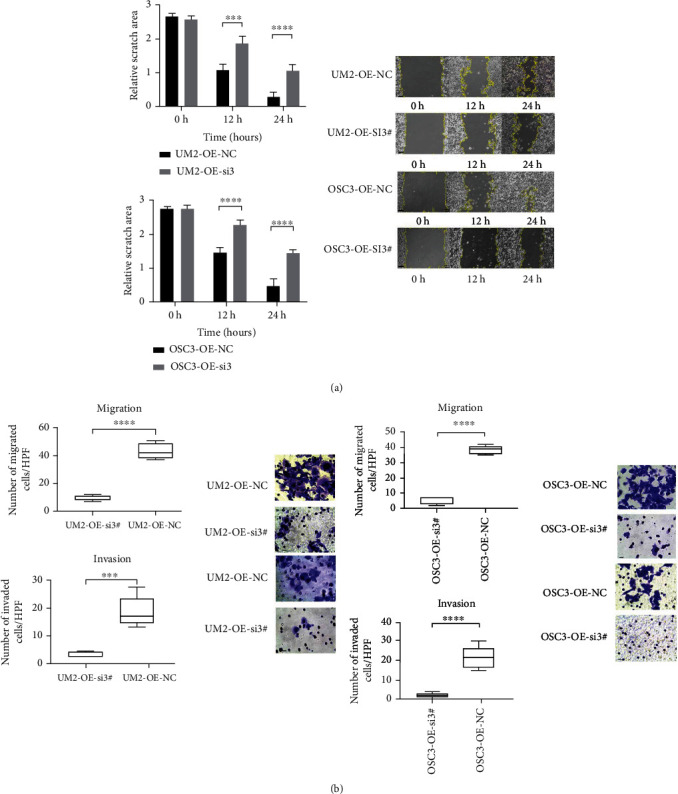
Silencing LEMD1 could rescue the phenotype in stably expressing LEMD1-AS1 OSCC cells. Wound healing assay in UM2 cell and OSC3 cell (a). Transwell assay in UM2 cells and OSC3 cells (b). si3#: LEMD1-si3#; NC: normal-control-si; OE: LEMD1-AS1-overexpressing. Scale bar in wound healing assay = 100 *μ*m. Scale bar in transwell assay = 20 *μ*m.

FISH assay showed that LEMD1-AS1 was mainly localized in the cytoplasm of OSCC cells, and minimal signals were observed in the nucleus (Additional Figure 4). To further analyze the biological role of LEMD1-AS1 in OSCC cells, we knocked down its expression in the OSC19 and CAL27 cells using the smart silencer (Additional Figure 5A), and ectopically expressed it in the UM2 and OSC3 cells (Additional Figure 5B). The cells overexpressing LEMD1-AS1 had higher levels of LEMD1, while LEMD1-AS1 knockdown was associated with downregulation of LEMD1 (Additional Figure 5C-D). Neither LEMD1-AS1 silencing nor overexpression had any effect on the proliferation of the OSCC cells compared to the respective controls (Additional Figure 6). However, LEMD1-AS1 overexpression in UM2 and OSC3 cells significantly increased their migration abilities *in vitro* (Figure 3), whereas LEMD1-AS1 knockdown had the opposite effect in OSC19 and CAL27 cells. Taken together, LEMD1-AS1 is a prometastatic factor in OSCC. To determine whether LEMD1-AS1 mediated its effects in OSCC via LEMD1, we knocked down the latter in cells stably overexpressing LEMD1-AS1 (Additional Figure 7). As shown in Figure 4, the knockdown of LEMD1 abrogated the effects of LEMD1-AS1 overexpression on the migration and invasion abilities of OSCC cells. Thus, LEMD1-AS1/LEMD1 interaction is crucial for OSCC progression and metastasis.

### 2.6. LEMD1-AS1 Increased the Stability of LEMD1 mRNA by Forming a Protective RNA Duplex

**Figure 5:**
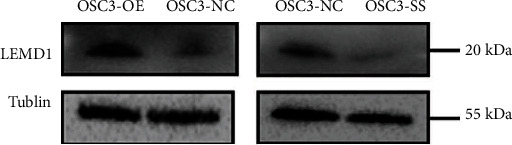
Western Blotting of LEMD1. After silencing LEMD1-AS1 for 48 hours in OSC3, the protein level of LEMD1 was diminished; and overexpression of LEMD1-AS1 increased LEMD1 expression at protein level. The samples were derived from the same experiment and that blots were processed in parallel. Full-length blots are presented in Supplementary Files.

**Figure 6:**
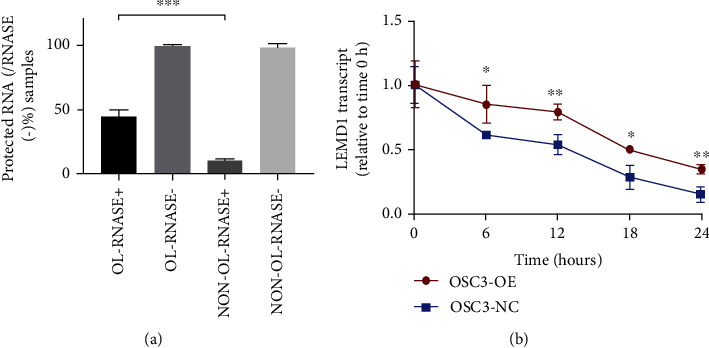
LEMD1-AS1 stabilized LEMD1 mRNA. (a) RNase protected assay indicated that the LEMD1-AS1 could protect OL region of LEMD1 from being depleted by RNase. (b) LEMD1-A1-overexpressing OSC3 cells were treated with 50 *μ*M *α*-amanitin, and the LEMD mRNAs were detected by qPCR. High levels of LEMD1-AS1 increased the stability of LEMD1 mRNA compared to that in control cells. 18sRNA was applied as an internal control.

The results so far indicated that LEMD1-AS1 regulated LEMD1 mRNA expression levels. Consistent with this, the LEMD1 protein levels were also increased in OSC3 cells stably overexpressing LEMD1-AS1 and decreased in LEMD1-AS1-knockdown cells (Figure 5). LEMD1-AS1 is localized at the antisense chain of the LEMD1 gene. In addition, antisense lncRNAs increase the stability of their cognate sense mRNAs by forming an RNA-RNA duplex, which also enhances the mRNA expression levels. Bioinformatics and gene sequence analysis revealed an overlapping (OL) region between LEMD1-AS1 and LEMD1. The RNase protection assay further showed that the remnant of the OL region between LEMD1-AS1 and LEMD1 was higher than the non-OL region, indicating that the OL region was partially protected from RNase degradation (Figure 6(a)). These results indicated that the stability of LEMD1 mRNA was increased by LEMD1-AS1. To functionally validate this surmise, we treated control or LEMD1-AS1-overexpressing OSC3 cells with the RNA polymerase II inhibitor *α*-amanitin to block new RNA synthesis and found that high levels of LEMD1-AS1 increased the stability of LEMD1 mRNA compared to that in control cells (Figure 6(b)). Thus, LEMD1-AS1 stabilizes and enhances the expression of LEMD1 mRNA in OSCC cells.

### 2.7. LEMD1-AS1 Activates the PI3K-AKT Pathway

On the basis of bioinformatics analysis and literature review, we analyzed the level of PI3K-AKT pathway-related proteins in the LEMD1-AS1-overexpressing OSCC cells. As shown in Additional Figure 8, p-PI3K and p-AKT were significantly upregulated in the OSC3 cells stably overexpressing LEMD1-AS1 compared to the control cells. Thus, LEMD1-AS1 might activate the PI3K-AKT pathway via increasing LEMD1 mRNA and protein levels.

### 2.8. LEMD1-AS1 Promoted Cervical LN and Hepatic Metastasis of OSCC *In Vivo*

**Figure 7:**
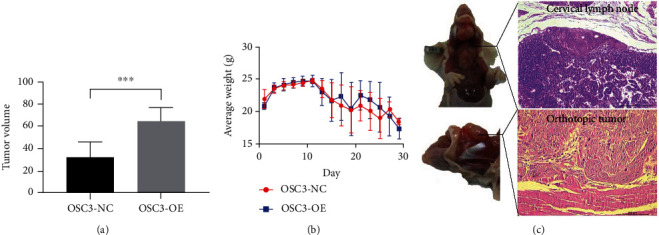
(a) The volume of the orthotopic tumor was markedly larger in the OSC3-OE compared to the OSC3-NC group. (b) However, there was no significant difference of weight between two groups. (c) Histochemical images of the orthotopic tumor and the metastatic lymph nodes.

To confirm the biological function of LEMD1-AS1 *in vivo*, we established cervical LN and hepatic metastasis models in B/C mice. LEMD1-AS1-overexpressing (OSC3-OE) or normal control (OSC3-NC) OSCC cells were injected into the mice FOM, and while 37.5% of the OSC3-OE mice had cervical LN metastasis, the OSC3-NC group did not show any metastasis (Additional Figure 9). Contradictory to the *in vitro* results, the volume of the orthotopic tumor was markedly larger in the OSC3-OE versus the OSC3-NC group, implying a greater proliferative capacity of the OSC3-OE cells *in vivo* (Figure 7).

## 3. Discussion

Recent studies have associated aberrant expression levels of lncRNAs with OSCC genesis and progression. However, little is known regarding the function of dysregulated lncRNAs in OSCC with regional LN metastasis [13, 14], which is the most pervasive cause of death in OSCC patients. To determine the role of lncRNAs and their target genes in cervical LN metastasis of OSCC, we identified the differentially expressed mRNAs and lncRNAs between primary OSCC samples with and without regional LN metastasis, since the expression pattern of primary tissues was similar to that of metastatic tissues according to the single cell sequencing result of head and neck squamous cell carcinoma in 2017 [15]. The DERNAs were enriched in GO components and KEGG pathways associated with tumor progression, migration and invasion, such as cell adhesion [16, 17], channel and receptor activity [18–20], cAMP signaling pathway [21], PI3K-AKT signaling pathway [22, 23], cytokine-cytokine receptor interaction, and CAMs [24]. The DElncRNA-DEmRNA network was subsequently constructed, and LEMD1-AS1 and its antisense mRNA LEMD1 were identified as a relevant pair in OSCC. LEMD1 [25] is a member of cancer–testis antigen (CTA) family and is located at chromosome 1q32.1. LEMD1-AS1 and LEMD1 were both upregulated in OSCC with LN metastasis compared to the nonmetastatic samples, indicating that LEMD1 and its reverse chain LEMD1-AS1 might enhance the migration and invasion abilities of OSCC cells.

Although LEMD1-AS1 gain/loss of function had no effect on OSCC cell growth, the LEMD1-AS1-overexpressing OSC3 cells resulted in larger tumors compared to the control cells. This was likely due to the fact that increased invasiveness of these cells led to impingement of the orthotopic tumor into the mandibula and FOM muscle, which resulted in larger tumor volume. In the orthotopic OSCC model as well, OSC3-OE cells resulted in higher LN and hematogenous metastasis rates, which further verified the metastatic potential of LEMD1-AS1. Consistent with this, LEMD1-AS1 significantly promoted OSCC migration and invasion *in vitro*. Furthermore, LEMD1 silencing neutralized the pro-metastatic effects of LEMD1-AS1, indicating that LEMD1-AS1 directly targeted LEMD1 to increase OSCC cell invasiveness.

More than 63% of all transcripts in human cells possess antisense transcripts, which upon any perturbation can alter the expression of sense mRNAs [26–28]. Studies increasingly show that natural antisense lncRNA can stabilize its counterpart mRNA to increase its expression levels [29–31]. RNA-asRNA interactions are the result of the formation of RNA-RNA hybrid, partial physical binding [32, 33] or activating polysomes [27]. Bioinformatics analysis revealed that LEMD1-AS1 and LEMD1 formed a “tail-to-tail” paring pattern with a 183 bp OL region. In addition, LEMD1-AS1 was localized in the cytoplasm, indicating the possibility of duplex formation between LEMD1 and its antisense lncRNA. Furthermore, the OL region on LEMD1 mRNA was protected from RNase digestion, which depleted most of the non-OL region. Finally, overexpression of LEMD1-AS1 increased stability of LEMD1 mRNA even in the presence of the RNA polymerase II inhibitor *α*-amanitin. Thus, LEMD1-AS1 can stabilize LEMD1 mRNA and protect it from RNase via RNA-RNA interaction.

CTAs are upregulated in male germ cells and various cancer tissues, but not in normal tissues [34]. This protein cluster promotes epithelial mesenchymal transition (EMT) [35] and metastasis [36], invasion, and carcinogenesis. Not surprisingly, CTAs are attractive diagnostic biomarkers and therapeutic targets in cancer. LEMD1 also promotes the initiation and progression of various cancers like colorectal cancer [25, 37, 38] and prostate cancer [39]. Sasahira et al. [40] identified LEMD1 as a novel oncogene in OSCC and supported its diagnostic and therapeutic potential. LEMD1 is also the target gene of microRNA-135 in anaplastic large cell lymphoma [41]. We have elucidated the regulatory interaction between LEMD1-AS1 and LEMD1 for the first time, which provides novel insights into the mechanism of CTAs in cancer.

The PI3K-AKT pathway is crucial for cancer initiation and progression, and is frequently disrupted in solid tumors [22, 42]. Mutation or alterations in the PI3K-AKT pathway have been identified in OSCC [24, 43–45]. Consistent with a previous study in gastric cancer [46], we found that the PI3K-AKT pathway was activated in OSC3 cells stably overexpressing LEMD1-AS1. We surmise therefore that LEMD1-AS1 upregulates LEMD1 to activate the PI3K-AKT pathway, which promotes OSCC migration and invasion.

Regional LN metastasis is the most common cause of the poor survival rate among OSCC patients. We identified 487 DEmRNAs and 1507 DElncRNAs in the metastatic versus nonmetastatic OSCC samples and characterized LEMD1-AS1/LEMD1 interaction as a promoter of OSCC metastasis for the first time. Mechanistically, LEMD1-AS1 activates the PI3K-AKT pathway by stabilizing and upregulating LEMD1. However, survival analysis related to LEMD1-AS1 expression was not possible due to the short follow-up time. In addition, the exact regulatory axis between LEMD1-AS1, LEMD1, and PI3K-AKT pathway in OSCC progression remains to be elucidated. Besides, the targeted genes of LEMD1-AS1 might not be only LEMD1; the relationship between its targeted genes should be further investigated. Our findings have to be validated on larger cohorts with longer follow-up.

## 4. Conclusions

LEMD1-AS1 was substantially increased in metastatic OSCC tissues and cell lines and promoted OSCC migration and invasion *in vitro* and *in vivo* by stabilizing its antisense transcript LEMD1, which is a potential activator of the PI3K-AKT signaling pathway. Therefore, LEMD1-AS1 is a novel diagnostic biomarker and immunotherapeutic target for metastatic OSCC.

## 5. Methods

### 5.1. Human Tissue Samples

Tumor tissues were collected from OSCC patients who underwent surgery at the Xiangya Hospital of Central South University. The patients that received radiotherapy or chemotherapy prior to the surgery were excluded. The baseline data of recruited patients is present in Table 1. All primary tumor tissue samples were confirmed by two experienced pathologists and stored at -80°C for RNA extraction. The clinical characteristics of the included patients were also recorded. The informed consent was obtained from all subjects. This study was approved by the ethics committee of the Xiangya Hospital (No. 201907790).

### 5.2. Next-Generation RNA Sequencing and Bioinformatics Analysis

Total RNA was isolated from 5 patients with cervical LN metastasis and 5 patients without cervical LN metastasis using the TRIzol reagent (Invitrogen, CA, USA) according to the manufacturer's instructions. The quality of RNA was evaluated by Qubit, Nanodrop, and Agilent 2100 Bioanalyzer. RNA sequencing libraries were prepared using TruSeq Stranded Total RNA Library Prep Kit according to manufacturer's specifications. The rRNAs were then removed, and the remaining transcripts were purified and fragmented. First-strand cDNA synthesis was performed using random primers, followed by second-strand cDNA synthesis and end repair. The 3′ ends of the cDNAs were adenlylated and ligated to Illumina Truseq adaptors for PCR. The cDNA libraries were sequenced by Illumina Hiseq 2500. All bioinformatical analysis were performed by R and LncTar software. *P* value was adjusted for multiple testing adopting the false discovery rated method, and |log2 fold change (FC)| > 1.0 and *P* value< 0.05 were set as the cutoff criteria.

### 5.3. OSCC Cell Lines and Animals

OSCC cell lines including UM1, UM2, OSC3, OSC19, and CAL27 were cultured in high-glucose DMEM (Gibco, CA, USA) supplemented with 10% fetal bovine serum (Gibco, CA, USA), 100 U/ml penicillin, and 100 mg/ml streptomycin (Gibco, CA, USA) at 37% in a humidified incubator containing 5% CO_2_. BALB/c-nude mice (5-week-old) were obtained from the Experiment Animal Center of Central South University. All animal experiments were approved by the Institutional Animal Care Committee of Central South University (No.2019sydw0116).

### 5.4. qRT-PCR Analysis

Total RNA was isolated using TRIzol reagent (Invitrogen, CA, USA) as described above, and reverse transcribed to cDNA by HiScript III RT SuperMix (Vazyme, Nanjing, China). Real-time PCR was performed on QuaintStudio 7 Flex System (Thermo Fisher, CA, USA) using the SYBR All-in-One qPCR mix (GeneCopoeia, Guangzhou, China), and relative gene expression was calculated using 2^–*ΔΔ*CT^ method normalized to that of GAPDH or 18sRNA. Primer sequences are listed in Table 2.

### 5.5. Fluorescent in Situ Hybridization

RNA fluorescence in situ hybridization (RNA-FISH) was performed using a FISH kit (Ribobio, Guangzhou, China) according to the manufacturer's instruction. Briefly, the suitably treated cells were fixed with 4% formaldehyde for 10 min, permeabilized with 0.5% Triton X-100 in PBS, and subsequently blocked through prehybridization at 37°C for 30 minutes. The cells were then incubated overnight with 50 nM FISH probe (Ribobio Co.) in 100 *μ*l hybridization buffer at 37°C. The slides were washed with a gradient of hybridization wash buffer (4 × SCC with 0.1% Tween-20, 2 × SCC, 1 × SCC) at 42°C for 5 min, respectively, and air-dried. After counterstaining with 4′,6-diamidino-2-phenylindole (DAPI), the slides were imaged under a fluorescence microscope.

### 5.6. Transfection

The Ribo™ Smart Silencer targeting LEMD1-AS1 was obtained from RiboBio (Guangzhou, China) and included three siRNA and three antisense oligonucleotides targeting different sequences. The siRNAs for human LEMD1 were designed and synthesized by RiboBio (China). Cells were transfected with the respective siRNAs using Lipofectamine3000 Reagent (Invitrogen, CA, USA). In addition, OSCC cells were transduced with LEMD1-AS1 expressing lentivirus (GENECHEM, Shanghai, China) with specific MOI (multiplicity of infection). The siRNA sequences are listed in Table 3.

### 5.7. CCK-8 Assay

Cell growth was monitored using the 2-(2-methoxy-4-nitrophenyl)-3-(4-nitrophenyl)-5-(2,4-disulfothenyl)-2H-tetrazolium reagent (CCK-8, Meilunbio, Dalian, China) assay according to manufacturer's instruction. The transient and stable transfectants were seeded in 96-well plates at the density of 3000 cells/well with 5 replicates per sample. CCK-8 reagent was added to each well after 1, 2, and 3 days of culture, and the absorbance of each well was measured at 450 nm.

### 5.8. Wound Healing Assay

Suitably treated cells were seeded in six-well plates and grown till confluency. The monolayer was scratched across the plate using a sterile 10 *μ*l pipette tip, and the dislodged cells were washed. The wounded regions were photographed at 0, 12, and 24 hours after scratching, and the scratch area was calculated by ImageJ software.

### 5.9. Transwell Assay

Cells harvested 36~48 hours after transient or stable transfection were seeded into the upper chambers of a transwell insert (Corning (3422, 354480), NY, USA) at the density of 8 × 10^4^ cells/well in 200 *μ*l serum-free high-glucose DMEM. The lower chambers were filled with 400 *μ*l DMEM supplemented with 20% FBS. After a 24 h culture, the migrated cells were fixed with 4% paraformaldehyde for 30 minutes, stained with crystal violet for 30 minutes, and counted.

### 5.10. Western Blotting

Protein was extracted by lysing the cells in RIPA buffer (Abcam, NY, USA) supplemented with protease inhibitors and phosphate inhibitors, separated by SDS-PAGE and transferred onto a PVDF membrane. After blocking in 5% skimmed milk for 1 hour, the gels were incubated overnight with the primary antibodies (Abcam, MA, USA and CST, MA, USA), followed by the HRP-conjugated IgG secondary antibody. The bands were visualized using ECL Substrates (SAB, MD, USA). Tubulin was used as the loading control.

### 5.11. RNase Protection Assay

RNA duplex formation between LEMD1-AS1 and LEMD1 was assessed with the RNase A+T cocktail (Invitrogen, CA, USA) that can only digest single-stranded and not duplex RNAs. Briefly, the samples were incubated with the enzyme cocktail at 37°C for 30 minutes, and the remaining double-stranded RNA was extracted using RNeasy Kit (Tianmo, Beijing, China) and analyzed by qRT-PCR.

### 5.12. Stability and *α*-Amanitin Treatment

OSC3 cells stably expressing LEMD1-AS1 or the empty vector were seeded into 6-well plates and treated with 50 *μ*M of the RNA synthesis blocker *α*-amanitin. The cells were harvested 0, 6, 12, 18, and 24 hours posttreatment and analyzed by qRT-PCR. The 18s RNA was used as the internal control since it is stable after *α*-amanitin treatment.

### 5.13. Animal Experiments

An orthotopic oral tumor model was established in mice by injecting control or LEMD1-AS1-overexpressing 2 × 10^5^ OSC3 cells (*n* = 8 per group) in 100 *μ*l DMEM into the floor of mouth (FOM) via submental to the space between the FOM muscles (around 5 mm). The mice were sacrificed on day 30 after implantation or when their weight was reduced to 16 grams (g) or less. The tongue and cervical LNs were collected and fixed in 10% paraformaldehyde immediately for HE staining. The volume of the orthotopic tumor was calculated as length × width^2^/2. All tissue staining results were examined by two expert pathologists.

### 5.14. Statistical Analysis

All data were analyzed using the SPSS 22.0 statistical software package (SPSS Inc., Chicago, IL, USA) and visualized with Graphpad Prism 7. The results were expressed as the mean ± SD of three experiments. Two or multiple groups were compared using two-tailed Student's *t*-test and One-way ANOVA, respectively. Pearson's correlation coefficient was used to analyze the correlation between LEMD1-AS1 and LEMD1 expression levels. *P* value < 0.05 was considered statistically significant.

## Figures and Tables

**Table 1:** Baseline data of the patients recruited.

No.	Gender	Age (year)	Location	TNM stage	Differentiation level	Drinking	Smoking (*n*/day)
1	Male	52	Tongue	T1N2M0	High	+	10-20
2	Male	29	Tongue	T1N2M0	Moderate-high	—	<5
3	Male	50	Tongue	T1N1M0	High	+	0
4	Male	39	Bucal	T1N1M0	Moderate-high	+	10-20
5	Male	48	Tongue	T1N1M0	High	+	0
6	Male	52	Buccal mucosa	T3N0M0	High	—	10-20
7	Male	65	Tongue	T3N0M0	High	+	10-20
8	Male	47	Buccal mucosa	T3N0M0	High	+	10-20
9	Male	55	Buccal mucosa	T3N0M0	Moderate-high	—	<5
10	Male	55	Tongue	T3N0M0	High	+	<5

**Table 2:** Primers used for qRT-PCR.

Gene	Forward primer	Reverse primer
LEMD1-AS1	TGCAGCTCAGTCAGACCAAA	AGGCAGACGTGGGAGGAT
LEMD1	GAGCACCAAGCACCAGAATCA	ACCAAGCACAGCAAGCTTCA
LEMD1-OL	TGGACCCAGAGAGCTGGATG	TGCGTTTAGTGGTGGAAGCC
LEMD1-non-OL	ACTTCTATCATCATGGTGGATG	GATCTGTGAGAGCAGCACAG
GAPDH	AGTGGTCGTTGAGGGCAAT	GCATCCTGGGCTACACTGAG
18sRNA	GTAACCCGTTGAACCCCATT	CCATCCAATCGGTAGTAGCG
PURPL	GGCATGATCTCGGCTCACTA	CAGATCACGAGGTCAGGAGA
TBILA	TGACTTTCAAAGCACAGGAGG	CCATGATTCCTGTCCCGAGA
LINC01133	TGGTATTTTCATCATTGTGGTGT	TCAGGGTAGTGTTTTGGTTCTTT

**Table 3:** Sequences of Smart Silencer for LEMD1-AS1 and siRNA for LEMD1.

Name	Targeted Sequence (5′-3′)
LEMD1-AS1 Smart Silencer	GACCAAACCTCTCTGAATA GACAGAACAAGAAGCACAA CTCTACATATCCATCACAT TCCAGCGCCCACTTTCTCAG GTCAGGACACAACAATAGAG ACAGCTCCTAGGCAATCAAA
LEMD1-siRNA1#	GAATCACATATGGGACTAT
LEMD1-siRNA2#	CGGAAGACCAGACTATCGA
LEMD1-siRNA3#	GCTGGAGAGAAGAAGGTTT

## Data Availability

The data used to support the findings of this study are included within the article and the supplementary information files.

## Supplementary Materials

Supplementary Materials Additional Figure 1: functional enrichment analysis of DEmRNAs. (A) The bar plot of GO analyses. Purple for MF, orange for CC, and yellow for BP. (B) The top 10 enrichment scores in KEGG pathway. Additional Figure 2: DElncRNA-DEmRNA interaction network in *cis*- and *trans*-way. Circles indicate mRNAs, and rectangles indicate lncRNAs. Red nodes mean upregulation in metastatic OSCC samples, while green nodes represent downregulation. (A) *Cis*-way. (B) *Trans*-way. Additional Figure 3: expression of LEMD1-AS1 in OSCC cell lines. Five OSCC cell lines were examined. The metastatic OSCC cell lines UM1, OSC19, and CAL27 showed significantly higher LEMD-AS1 levels compared to the nonmetastatic UM2 and OSC3 cells. The red ∗ indicated statistical difference with OSC3, and blue one indicated that with UM1. Additional Figure 4: subcellular location of LEMD1-AS1. Fish assay revealed that LEMD1-AS1 mainly is located in the cytoplasm, while a few in the nucleus. Red shows LEMD1-AS1, and blue shows nucleus; scale bar = 50 *μ*m. Additional Figure 5: expression of LEMD1AS1 and LEMD1 in transfectants. (A) LEMD1-AS1 knockdown efficiency in OSC19 and CAL27 with Smart Silencer. (B) LEMD1-AS1 overexpression efficiency in UM2 and OSC3 with lentivirus. (C) LEMD1 mRNA expression level was decreased in LEMD1-AS1-knockdown OSCC cells. (D) LEMD1 mRNA expression level was elevated in LEMD1-AS1-overexpressing OSCC cells. Additional Figure 6: CCK8 assay implied that LEMD1-AS1 was not able to influence the cell growth in OSCC cells. (A) Knockdown of LEMD1-AS1 in OSC19 and CAL27 cells did not affect cell growth. (B) Overexpression of LEMD1-AS1 in UM2 and OSC3 cells did not change the ability of growth. SS: LEMD1-AS1 Smart Silencer; NC: normal control; OE: LEMD1-AS1-overexpressing. Additional Figure 7: apply 3 sequences of siRNA to inhibit LEMD1 expression. Among these, si3# had the highest transfection efficiency. Additional Figure 8: Western Blotting showed that the level of PI3K-AKT pathway-related proteins was increased in LEMD1-AS1-overexpressing OSC3 cells compared to the control cells. Additional Figure 9: the metastasis ratio of two groups (LEMD1-AS1-overexpressing and normal control (NC)).
