# Supplementary material for: Long Noncoding RNA LEMD1-AS1 Increases LEMD1 Expression and Activates PI3K-AKT Pathway to Promote Metastasis in Oral Squamous Cell Carcinoma
Source: Biomed Res Int. 2022 Aug 9;2022:3543948. doi: 10.1155/2022/3543948 (PMC9381283; doi:10.1155/2022/3543948)
Supplement: Supplementary Materials — Additional Figure 1: functional enrichment analysis of DEmRNAs. (A) The bar plot of GO analyses. Purple for MF, orange for CC, and yellow for BP. (B) The top 10 enrichment scores in KEGG pathway. Additional Figure 2: DElncRNA-DEmRNA interaction network in cis- and trans-way. Circles indicate mRNAs, and rectangles indicate lncRNAs. Red nodes mean upregulation in metastatic OSCC samples, while green nodes represent downregulation. (A) Cis-way. (B) Trans-way. Additional Figure 3: expression of LEMD1-AS1 in OSCC cell lines. Five OSCC cell lines were examined. The metastatic OSCC cell lines UM1, OSC19, and CAL27 showed significantly higher LEMD-AS1 levels compared to the nonmetastatic UM2 and OSC3 cells. The red ∗ indicated statistical difference with OSC3, and blue one indicated that with UM1. Additional Figure 4: subcellular location of LEMD1-AS1. Fish assay revealed that LEMD1-AS1 mainly is located in the cytoplasm, while a few in the nucleus. Red shows LEMD1-AS1, and blue shows nucleus; scale bar = 50 μm. Additional Figure 5: expression of LEMD1AS1 and LEMD1 in transfectants. (A) LEMD1-AS1 knockdown efficiency in OSC19 and CAL27 with Smart Silencer. (B) LEMD1-AS1 overexpression efficiency in UM2 and OSC3 with lentivirus. (C) LEMD1 mRNA expression level was decreased in LEMD1-AS1-knockdown OSCC cells. (D) LEMD1 mRNA expression level was elevated in LEMD1-AS1-overexpressing OSCC cells. Additional Figure 6: CCK8 assay implied that LEMD1-AS1 was not able to influence the cell growth in OSCC cells. (A) Knockdown of LEMD1-AS1 in OSC19 and CAL27 cells did not affect cell growth. (B) Overexpression of LEMD1-AS1 in UM2 and OSC3 cells did not change the ability of growth. SS: LEMD1-AS1 Smart Silencer; NC: normal control; OE: LEMD1-AS1-overexpressing. Additional Figure 7: apply 3 sequences of siRNA to inhibit LEMD1 expression. Among these, si3# had the highest transfection efficiency. Additional Figure 8: Western Blotting showed that the level of PI3K-AKT pathway-related protei [file 3543948.f1.docx]

**Additional Figures**


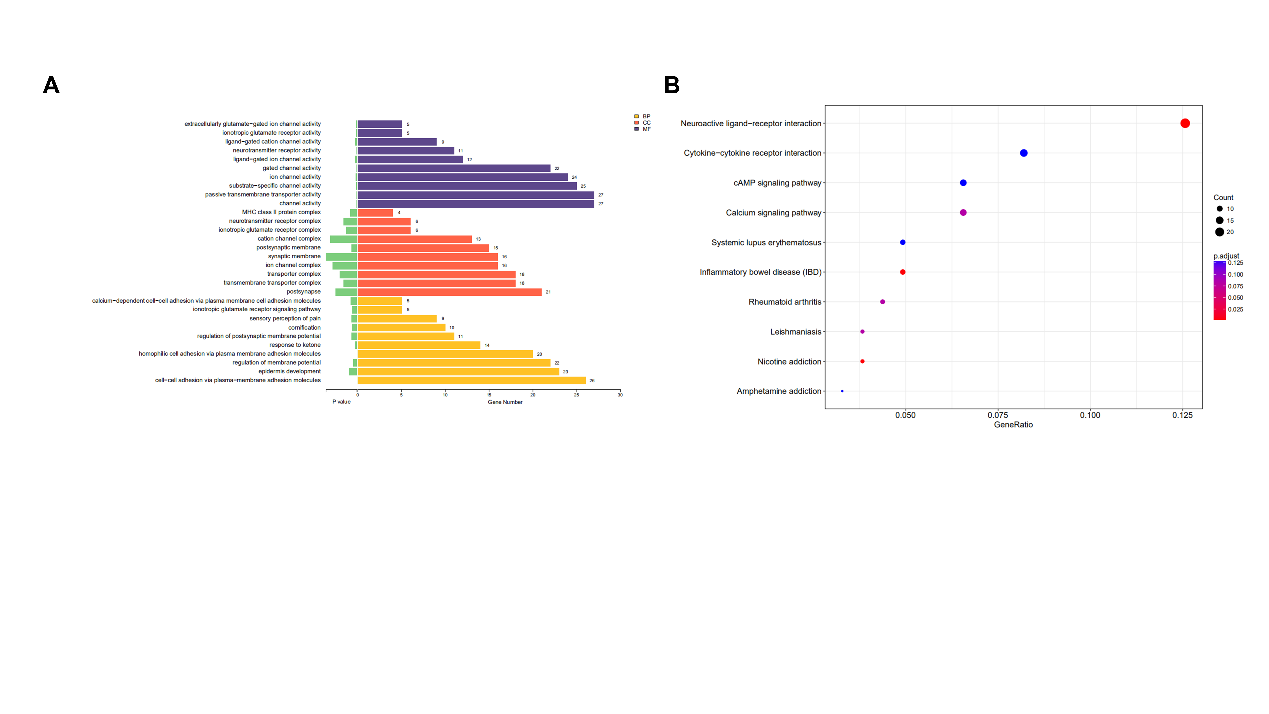


**Additional Figure 1**


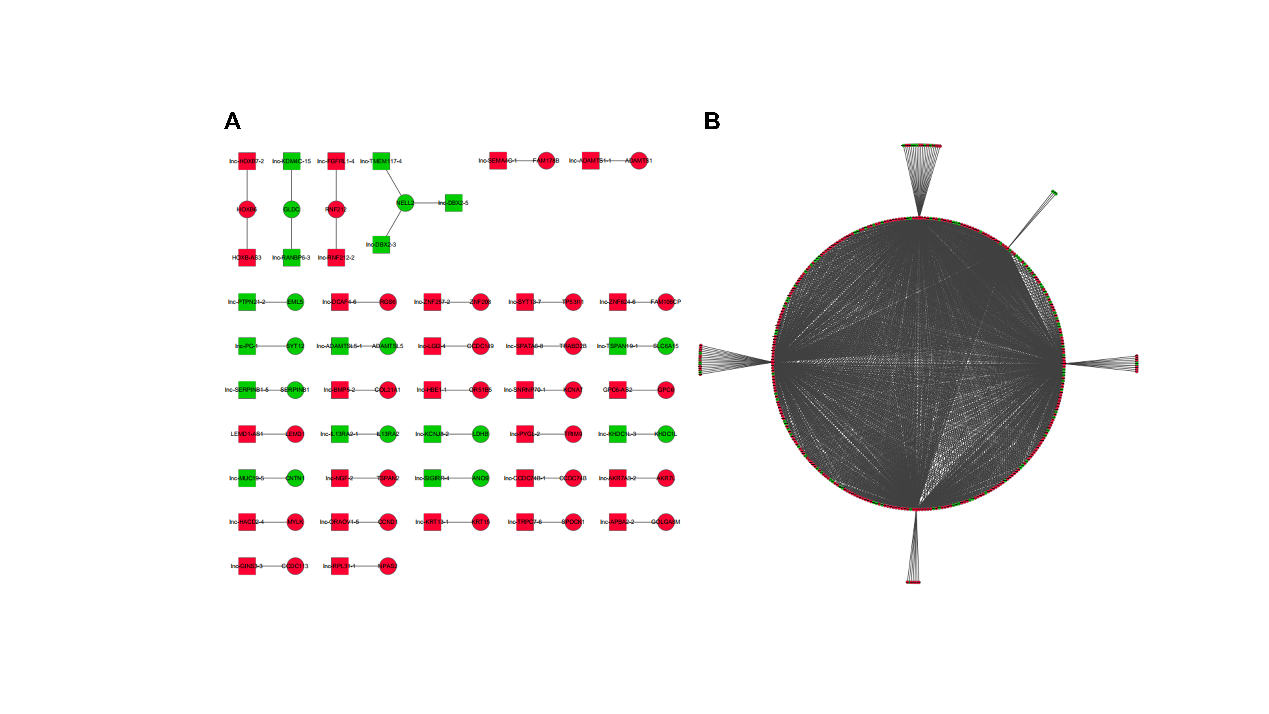


**Additional Figure 2**


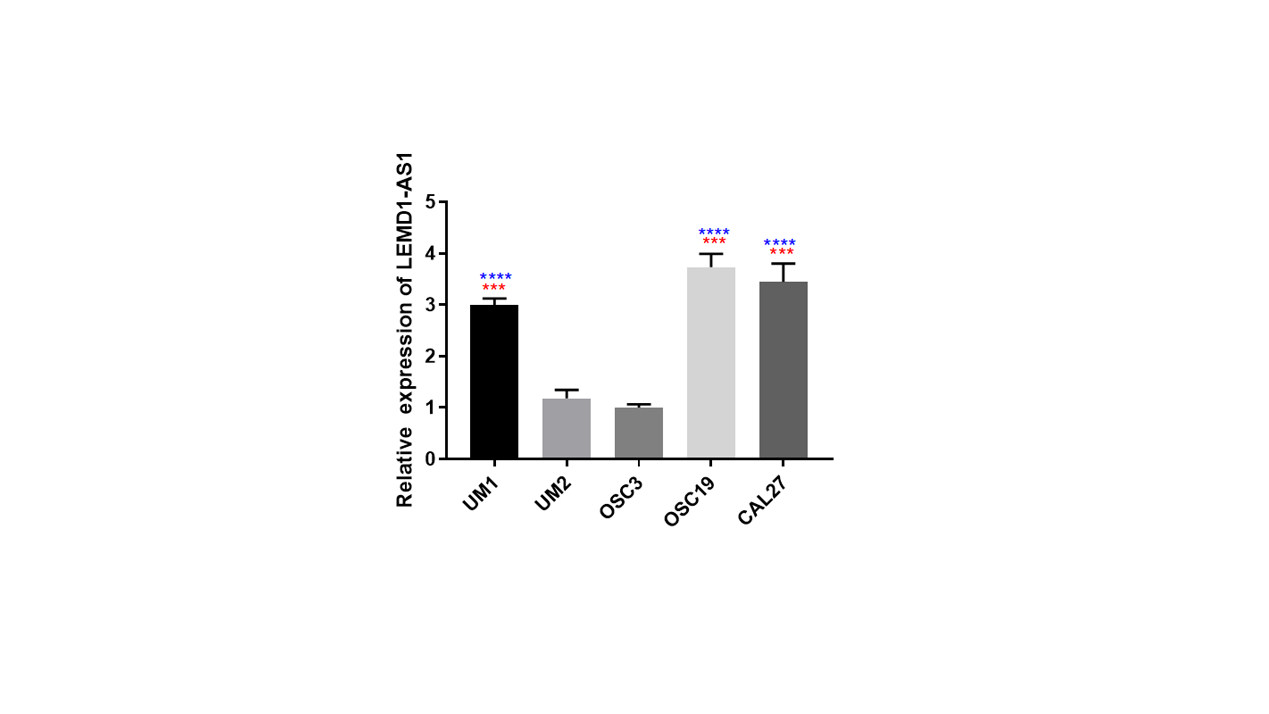


**Additional Figure 3**


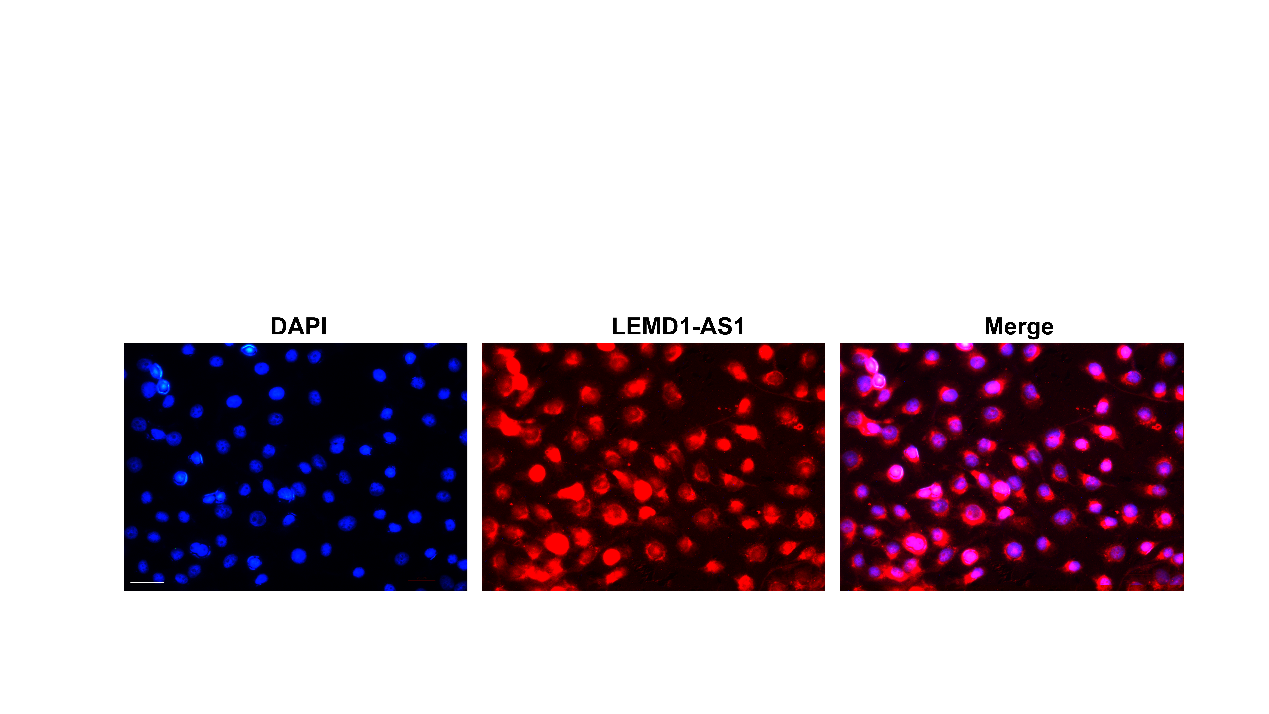


**Additional Figure 4**


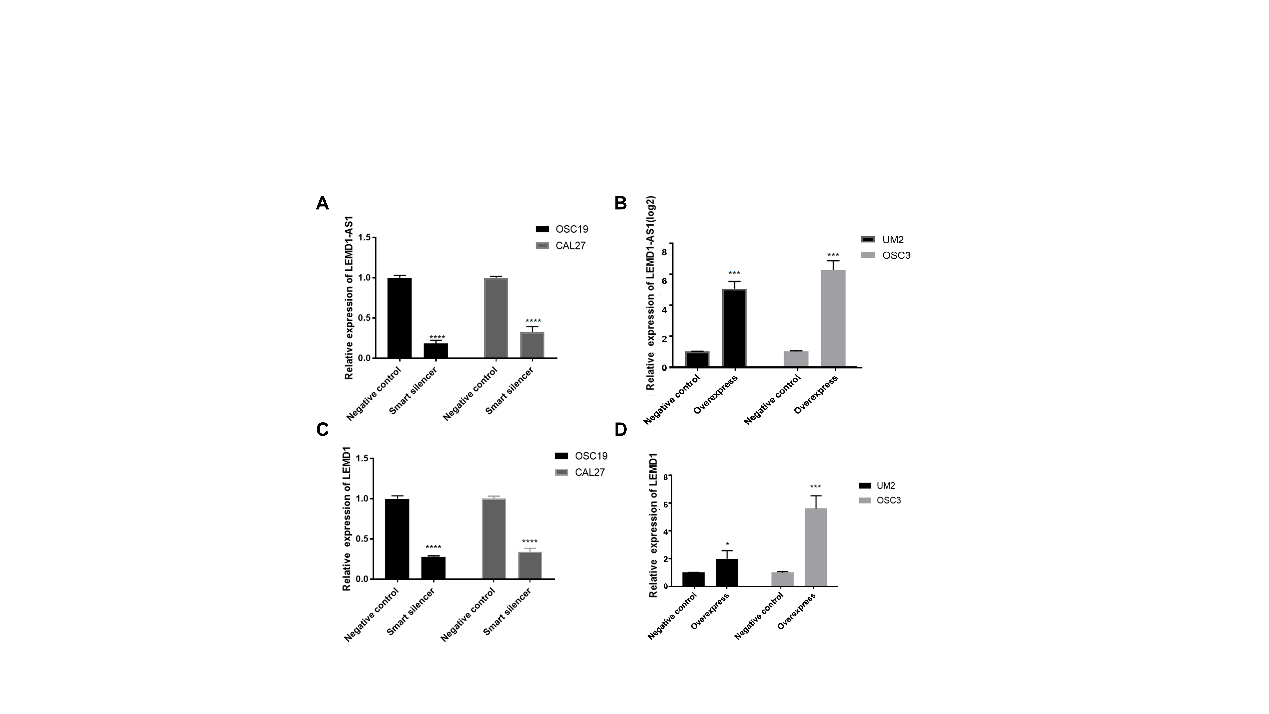


**Additional Figure 5**


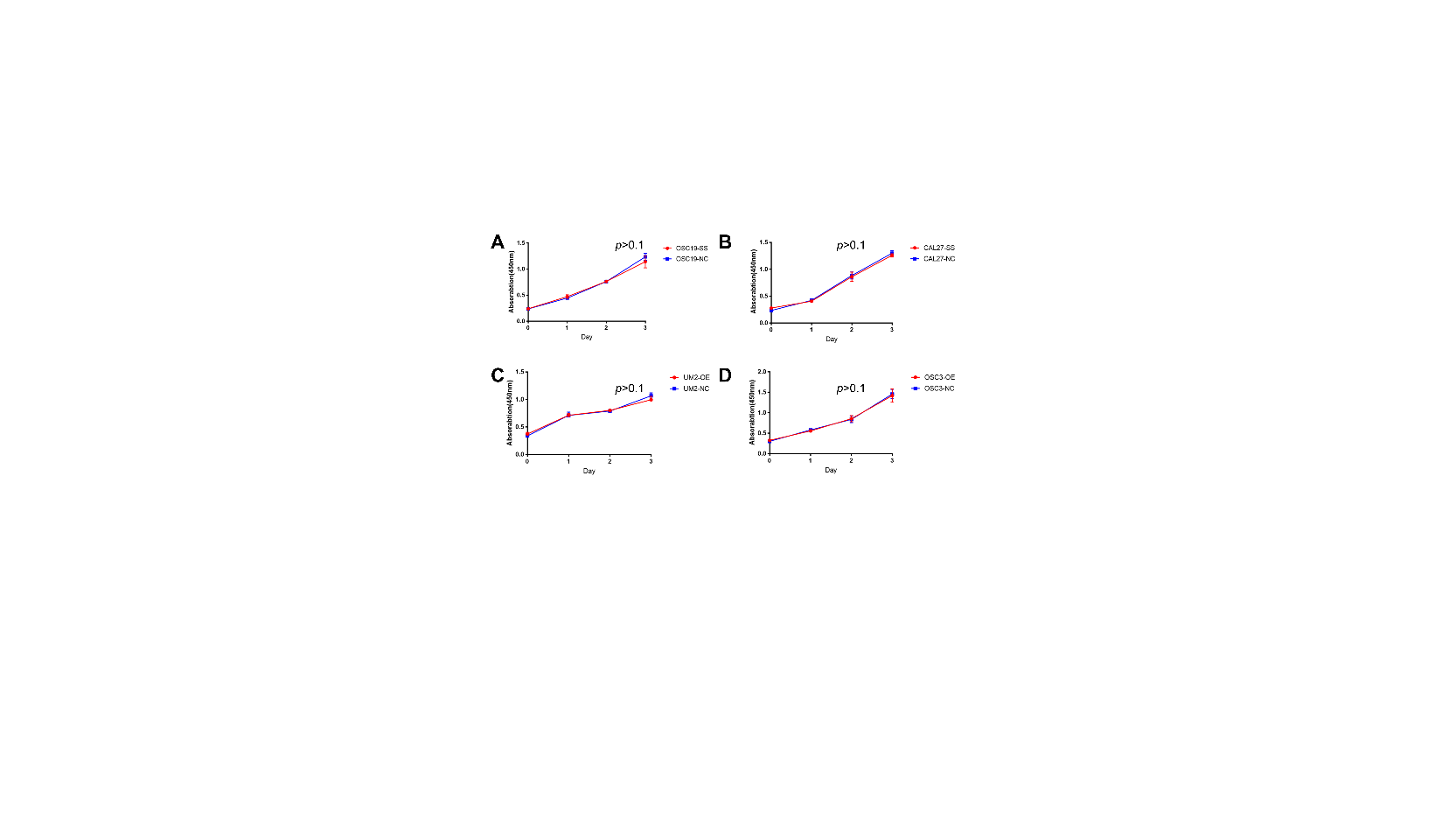


**Additional Figure 6**


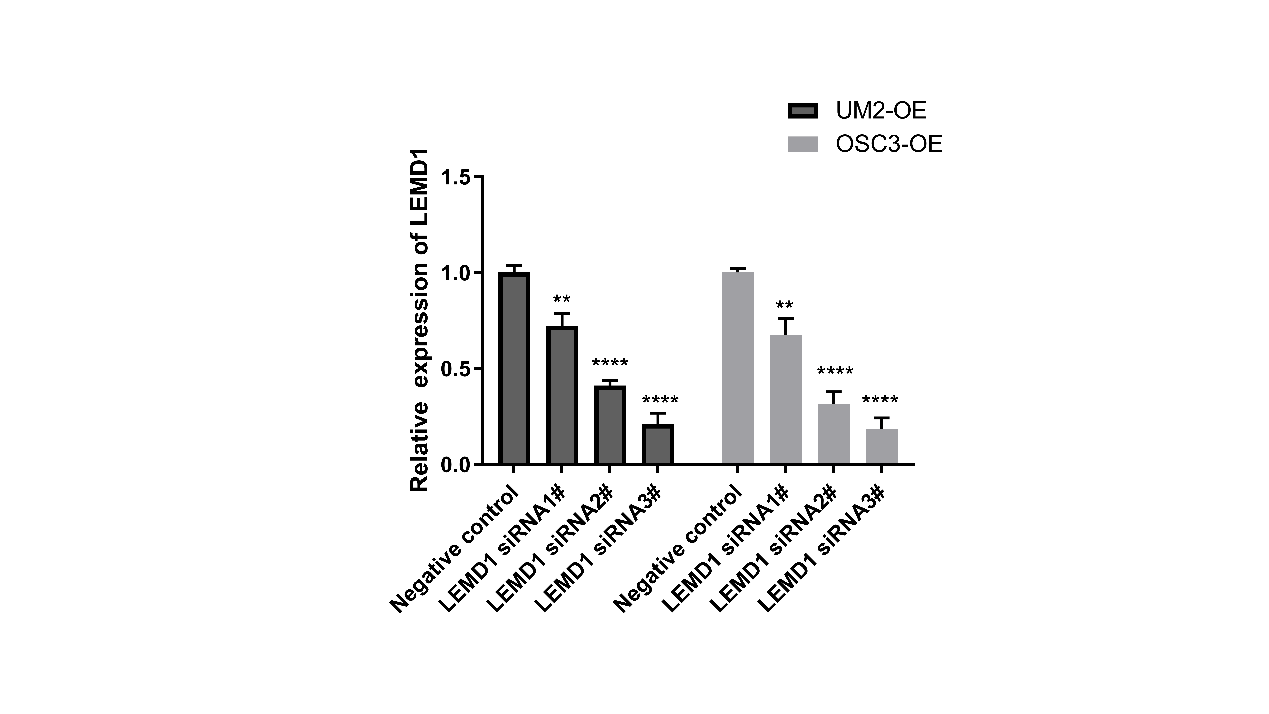


**Additional Figure 7**


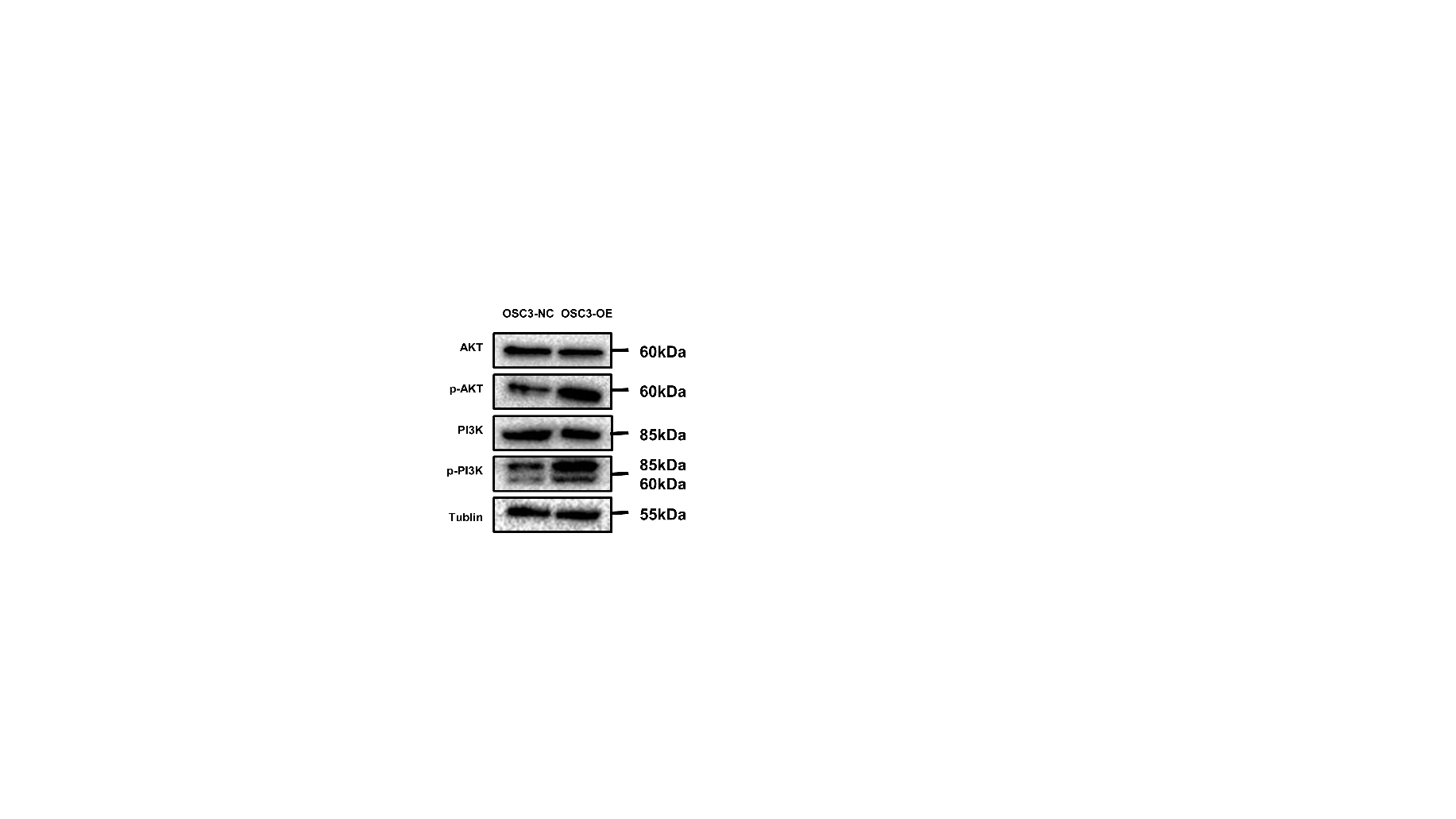


**Additional Figure 8**

**Additional Figure 9**
